# ChatGPT: Can You Prepare My Patients for [^18^F]FDG PET/CT and Explain My Reports?

**DOI:** 10.2967/jnumed.123.266114

**Published:** 2023-12

**Authors:** Julian M.M. Rogasch, Giulia Metzger, Martina Preisler, Markus Galler, Felix Thiele, Winfried Brenner, Felix Feldhaus, Christoph Wetz, Holger Amthauer, Christian Furth, Imke Schatka

**Affiliations:** 1 Department of Nuclear Medicine, Charité–Universitätsmedizin Berlin, Berlin, Germany;; 2 Berlin Institute of Health, Charité–Universitätsmedizin Berlin, Berlin, Germany;; 3 Charité Comprehensive Cancer Center, Charité–Universitätsmedizin Berlin, Berlin, Germany; and; 4 Department of Radiology, Charité–Universitätsmedizin Berlin, Berlin, Germany

**Keywords:** GPT-4, FDG PET/CT, artificial intelligence, chatbot, patient communication

## Abstract

We evaluated whether the artificial intelligence chatbot ChatGPT can adequately answer patient questions related to [^18^F]FDG PET/CT in common clinical indications before and after scanning. **Methods:** Thirteen questions regarding [^18^F]FDG PET/CT were submitted to ChatGPT. ChatGPT was also asked to explain 6 PET/CT reports (lung cancer, Hodgkin lymphoma) and answer 6 follow-up questions (e.g., on tumor stage or recommended treatment). To be rated “useful” or “appropriate,” a response had to be adequate by the standards of the nuclear medicine staff. Inconsistency was assessed by regenerating responses. **Results:** Responses were rated “appropriate” for 92% of 25 tasks and “useful” for 96%. Considerable inconsistencies were found between regenerated responses for 16% of tasks. Responses to 83% of sensitive questions (e.g., staging/treatment options) were rated “empathetic.” **Conclusion:** ChatGPT might adequately substitute for advice given to patients by nuclear medicine staff in the investigated settings. Improving the consistency of ChatGPT would further increase reliability.

## Visual Abstract

**Figure:**
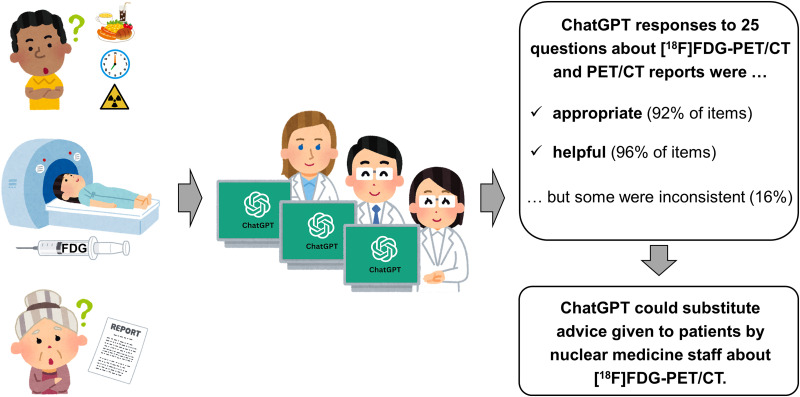

The use of PET/CT is expected to increase because of a growing awareness of its value in clinical decision-making (1). With limited staff resources and mounting individual workloads, there is a need to increase efficiency, such as through use of artificial intelligence (AI) (2). Specifically, large language models such as OpenAI’s generative pretrained transformer (GPT) 4 might represent an information tool for patients to answer their questions when preparing for an examination or when reviewing the subsequent report.

However, the reliability of GPT can be undermined by false and potentially harmful responses termed hallucinations (3,4). False responses occur less often with more advanced versions such as GPT-4 (5) but have still been observed by Lee et al. (3).

Within the discipline of nuclear medicine, Buvat and Weber recently reported a brief interview with the AI chatbot ChatGPT. While remaining cautious in providing recommendations or solutions, they found that ChatGPT could answer technical questions well (6). It is neither foreseeable nor desirable that AI tools will replace physicians for informed consent. Furthermore, use of such tools by nuclear medicine departments is currently limited by unsolved liability issues (7). However, if validated in a clinical context, such a tool might still be used by patients to obtain information and general advice currently given by nuclear medicine staff (mainly technologists and physicians) and thereby enhance patient compliance (8).

To our knowledge, ours was the first systematic investigation of ChatGPT (with GPT-4) for patient communications related to PET/CT with [^18^F]FDG. We evaluated whether ChatGPT provides adequate, consistent responses and explanations to questions frequently asked by patients.

## MATERIALS AND METHODS

### ChatGPT Responses

OpenAI ChatGPT Plus was used in the May 24 version of GPT-4 (https://openai.com/chatgpt). ChatGPT was accessed on May 25 and 26, 2023. All questions and PET/CT reports were entered as single prompts in separate chats. Each prompt was repeated twice using the regenerate-response function, resulting in 3 trials per prompt. In addition, ChatGPT was asked to provide references (19 tasks).

### Rating Process

Three nuclear medicine physicians, all of them native German speakers, rated the ChatGPT responses independently using the rating scale shown in Table 1. Appropriateness and usefulness were assessed with 4-point scales to prevent neutral responses and to facilitate binarization of results. Two readers were board-certified nuclear medicine physicians with more than 10 y of experience in PET/CT reading. The third reader was a resident in nuclear medicine with 2 y of PET/CT experience. The criterion “empathetic” was used only to rate the follow-up questions related to PET/CT reports. A binary item was used to avoid ambiguous or artificial grading with a multipoint scale.

**TABLE 1.** Criteria and Categories Used for Rating

Criterion	Description
Appropriateness	
1: Highly appropriate	Meeting standards of information given by medical staff in nuclear medicine department
2: Quite appropriate	Minor aspects incorrect or inconsistent
3: Quite inappropriate	Relevant aspects inconsistent
4: Highly inappropriate	Major aspects incorrect; potentially harmful
Helpfulness	
1: Very helpful	Comprehensive and likely to fully answer patient’s question
2: Quite helpful	Specific but lacking potentially helpful information
3: Quite unhelpful	Specific but lacking crucial information related to patient’s question
4: Clearly unhelpful	Unspecific and lacking crucial information
Empathetic	
Yes	Shows humanlike empathy
No	Is neutral and shows no empathy
Inconsistent between trials	
1: Irrelevant	Differences only in wording, style, or layout
2: Minor	Differences in content of response but none relevant to main content required to answer patient’s question
3: Major	Some differences relevant to main content
4: Incompatible	Responses incompatible with each other
Validity of references	
1: Fully valid	Appropriate, identifiable, and accessible source
2: Appropriate but outdated	Appropriate reference but outdated uniform resource locator or only generic references
3: Appropriate, incorrectly cited, but possible to find	Appropriate reference with incorrect bibliographic data but still possible to find
4: Invalid	Invalid reference that cannot be found (hallucinations)

In addition, 1 reader rated the level of inconsistency among the 3 responses generated for each question and checked and rated the validity of all references provided by ChatGPT.

### Generating Questions and PET/CT Reports

Thirteen questions frequently asked by patients concerning [^18^F]FDG PET/CT imaging (Table 2; Q1–Q13) were formulated using simple, nontechnical language (e.g., “PET scan”).

**TABLE 2.** All 25 Tasks Submitted to ChatGPT and Majority Rating

Question/report	Description	Appropriate	Helpful	Inconsistent
Q1	How long does a PET scan take?	1	1	2
Q2	Is a PET scan harmful?	1	1	1
Q3	How should I prepare for a PET scan?	1	1	2
Q4	I’m a caregiver to a toddler. Are there any precautionary measures after a PET scan?	3*	3*	3*
Q5	Can I take a PET scan as a diabetic?	1	1	1
Q6	Is a PET scan recommended for lung cancer before surgery?	2	2	2
Q7	Why is a PET scan needed for Hodgkin lymphoma?	1	1	1
Q8	How accurate is a PET scan for lung cancer?	1	2	1
Q9	Is a PET scan better than a CT scan for lung cancer?	2	1	1
Q10	Does negative on PET mean that a lung nodule is benign?	2	1	1
Q11	Does a hypermetabolic lung nodule on PET mean that I have lung cancer?	1	1	1
Q12	My PET scan showed a hypermetabolic lung nodule. Should I have it removed?	1	1	2
Q13	What does “Deauville 5” mean in a PET report?	1	1	1
R1	Hodgkin lymphoma: initial staging	1	1	2
R1Q1	What’s my lymphoma stage?	3*	2	3*
R2	Hodgkin lymphoma: response assessment	1	1	2
R3	NSCLC: initial staging (early stage)	1	1	1
R3Q1	How should my lung cancer be treated?	2	2	3*
R4	NSCLC: initial staging (locally advanced)	1	1	1
R4Q1	What’s my cancer stage?	2	1	3*
R4Q2	How should my lung cancer be treated?	1	2	2
R5	NSCLC: initial staging (stage IV)	1	1	1
R5Q1	What’s my life expectancy?	1	1	2
R6	Solitary pulmonary nodule (PET-negative)	1	1	1
R6Q1	Should the lung nodule be removed?	1	1	1

* Negative rating (category 3 or 4).

Inconsistent = only 1 rater.

Rating categories are explained in Table 1. In 5 of 6 PET/CT reports, follow-up questions were raised.

Five PET/CT reports (Table 2; R1–R5) were derived from fictitious reports based on templates from our institution. The German reports were first translated with DeepL and then edited. Additionally, a sample report, “Sample Normal Report #2—Negative SPN,” provided by the Society of Nuclear Medicine and Molecular Imaging (9) was used (R6). In R1–R6, the same prompt, “Please explain my PET report to me: [full text of the report],” was used. Since the PET/CT reports were fictitious, no ethical approval was needed.

### Statistical Analysis

The final rating for each task was selected by majority vote (except for “inconsistency” and “validity of references,” which were assessed by only 1 rater). When 3 different ratings arose, the middle category was chosen.

## RESULTS

All questions, PET/CT reports, and ChatGPT responses can be found in Supplemental Files 1 and 2 (supplemental materials are available at http://jnm.snmjournals.org).

### Rating of ChatGPT Responses

Responses by ChatGPT to 23 of 25 tasks were deemed “quite appropriate” or “fully appropriate” (92%, Table 2), whereas responses to 2 tasks (8%), R1Q1 and R4Q1, were rated “quite inappropriate.” Both questions queried tumor stage on the basis of a PET/CT report that did not explicitly state the tumor stage but contained information that would have enabled determining it using established staging systems. In both instances, ChatGPT identified 2 potential tumor stages, one of which was correct.

ChatGPT responses were rated “very helpful” or “quite helpful” by majority vote for 24 of 25 tasks (96%). The response to Q4 was rated “quite unhelpful” because ChatGPT did not caution against breastfeeding after a PET/CT scan, which might still be relevant for patients who are caretakers of toddlers.

In 5 of 6 follow-up questions (83%) related to the potential consequences of the PET/CT findings, ChatGPT responses were rated “empathetic.”

### General Observations

ChatGPT answers were structured so as to form intelligible responses. ChatGPT framed responses that are likely to cause emotional reactions such as anxiety in a reassuring way (e.g., when revealing an advanced stage of metastatic lung cancer [R4Q1]). This is one of the aspects that the raters regarded as general signs of natural and humanlike responses (Supplemental File 3; Supplemental Table 1).

When PET/CT reports were being explained, the level of certainty conveyed by the ChatGPT responses seemed to depend on the clarity and extent of interpretation given in the report itself. We did not observe responses that were unrelated to the specific content of the PET/CT reports (hallucinations).

In 1 response, ChatGPT was able to provide a correct interpretation when explaining the PET/CT report of metastatic lung cancer (R5), although this interpretation was not explicitly provided in the report (Supplemental File 3; Supplemental Table 1).

### Variation Among Trials

In responses to 21 of 25 tasks (84%), the 3 trials showed “irrelevant” or “minor” differences (Table 2). Responses to 4 tasks (16%)—3 of which were follow-up questions—were rated as showing “considerable” inconsistencies because ChatGPT addressed the specific tumor stage of the patient inconsistently.

### Validity of References

In 2 of the 19 tasks (11%), 1 reference was considered invalid (hallucination) because the article could not be found by a manual search (details in Supplemental File 3; Supplemental Table 2).

References were fully valid in only 4 of 19 investigated tasks (21%). In 11 tasks (58%), at least 1 reference contained an outdated uniform resource locator or was only generic (e.g., “National Institute of Health’s U.S. National Library of Medicine”). In responses to 2 of 19 tasks (11%), the referenced article could be found only via a manual search.

## DISCUSSION

None of the answers generated by ChatGPT would have caused harm or left the patient uninformed if the questions and PET/CT reports had been real patient inquiries.

Specifically, ChatGPT responses to more than 90% of questions were adequate and useful even by the standards expected of general advice given by nuclear medicine staff. In the 3 responses rated “quite unhelpful” or “quite inappropriate,” answers in at least one of the repeated trials were precise and correct. Although this observation shows that ChatGPT is per se capable of providing appropriate answers to all 25 tasks, this variation in responses led to a rating of “considerable inconsistency.” With future advances in AI models, the focus should be on reducing variation between responses so as to increase predictability and thus reliability.

The question of liability for AI-generated content still needs to be addressed. In a medical context, ChatGPT may be best regarded as an information tool rather than an advisory or decision tool. Every response from ChatGPT included a statement that the findings and their consequences should always be discussed with the treating physician (Supplemental File 3; Supplemental Table 3). Questions targeting crucial information, such as staging or treatment, were answered with the necessary empathy and an optimistic outlook.

We focused on the most common PET/CT tracer and on indications with a relatively large database of information available to GPT-4. The responses might be less helpful or reliable in the case of rare indications or new tracers, especially if the relevant literature has been published after the model training threshold (GPT-4: September 2021) (6). Validation in other contexts will therefore be required.

The issue of 2 invalid references to original articles that seem to have been hallucinated also demands further investigation.

## CONCLUSION

ChatGPT may offer an adequate substitute for informational counseling to patients in lieu of that provided by nuclear medicine staff in the currently investigated setting of [^18^F]FDG PET/CT for Hodgkin lymphoma or lung cancer. With ever-decreasing time available for communication between staff and patients, readily accessible AI tools might provide a valuable means of improving patient involvement, the quality of patient preparation, and the patient’s understanding of nuclear medicine reports. The predictability and consistency of responses from AI tools should be further increased, such as by restricting their sources of information to peer-reviewed medical databases.

## DISCLOSURE

No potential conflict of interest relevant to this article was reported.

## Notes

KEY POINTS **QUESTION:** Might ChatGPT substitute for advice given to patients on [^18^F]FDG PET/CT? **PERTINENT FINDINGS:** ChatGPT responses were appropriate and useful, but we observed some inconsistency between trials. **IMPLICATIONS FOR PATIENT CARE:** Proper use of AI tools might improve patients’ involvement and their understanding of PET/CT reports.
